# Repun: an accurate small variant representation unification method for multiple sequencing platforms

**DOI:** 10.1093/bib/bbae613

**Published:** 2024-11-25

**Authors:** Zhenxian Zheng, Yingxuan Ren, Lei Chen, Angel On Ki Wong, Shumin Li, Xian Yu, Tak-Wah Lam, Ruibang Luo

**Affiliations:** Department of Computer Science, The University of Hong Kong, Pok Fu Lam Road, Hong Kong, 999077, China; Department of Computer Science, The University of Hong Kong, Pok Fu Lam Road, Hong Kong, 999077, China; Department of Computer Science, The University of Hong Kong, Pok Fu Lam Road, Hong Kong, 999077, China; Department of Computer Science, The University of Hong Kong, Pok Fu Lam Road, Hong Kong, 999077, China; Department of Computer Science, The University of Hong Kong, Pok Fu Lam Road, Hong Kong, 999077, China; Department of Computer Science, The University of Hong Kong, Pok Fu Lam Road, Hong Kong, 999077, China; Faculty of Computing, Harbin Institute of Technology, 92 Xidazhi Street, Nangang District, Harbin, Heilongjiang 150001, China; Department of Computer Science, The University of Hong Kong, Pok Fu Lam Road, Hong Kong, 999077, China; Department of Computer Science, The University of Hong Kong, Pok Fu Lam Road, Hong Kong, 999077, China

**Keywords:** variant representation, representation unification, multiple platform sequencing, haplotype comparison, variant calling

## Abstract

Ensuring a unified variant representation aligning the sequencing data is critical for downstream analysis as variant representation may differ across platforms and sequencing conditions. Current approaches typically treat variant unification as a post-step following variant calling and are incapable of measuring the correct variant representation from the outset. Aligning variant representations with the alignment before variant calling has benefits like providing reliable training labels for deep learning-based variant caller model training and enabling direct assessment of alignment quality. However, it also poses challenges due to the large number of candidates to handle. Here, we present Repun, a haplotype-aware variant-alignment unification algorithm that harmonizes the variant representation between provided variants and alignments in different sequencing platforms. Repun leverages phasing to facilitate equivalent haplotype matches between variants and alignments. Our approach reduced the comparisons between variant haplotypes and candidate haplotypes by utilizing haplotypes with read evidence to speed up the unification process. Repun achieved >99.99% precision and > 99.5% recall through extensive evaluations of various Genome in a Bottle Consortium samples encompassing three sequencing platforms: Oxford Nanopore Technology, Pacific Biosciences, and Illumina. Repun is open-source and available at (https://github.com/zhengzhenxian/Repun).

## Introduction

Genetic variant identification plays a pivotal role in genetic research and clinical applications [1]. Several initiatives, such as the Genome in a Bottle Consortium (GIAB) [2] and Sequencing Quality Control Phase 2 [3], have developed small germline and somatic variant truths for well-characterized human genomes to establish golden standards for benchmarking. To facilitate the comparison and evaluation of variant calling results, comprehensive and standardized benchmarking methods are essential [4]. During the variant evaluation process, variants may exhibit diverse representations within the VCF format across various sequencing platforms, chemistries, and conditions. When comparing VCF outcomes record by record, the observed differences can merely arise from different representations of the same variant.

To address this issue, some variant calling performance evaluation frameworks are available, such as the RTG tool [5] introduced by Real Time Genomics, and hap.py [6] developed by the Global Alliance for Genomics and Health Team (GA4GH). These tools have introduced optimized searching algorithms to identify the most extensive matching subsets of query variants and truth variants, effectively determining the complete variant representation match [7]. However, these frameworks are designed for variant assessment and lack the capability to independently evaluate the variant representation within specific sequencing alignments without performing variant calling, primarily due to the vast number of candidates that need to be consolidated. Consequently, a universal method for variant representation unification is essential to address diverse sequencing data scenarios where variant caller is not always available.

Variant representation unification is the process of updating the variant representation to match the sequencing alignment while accurately representing the same haplotype with query variants. Some recent studies [8–11], have demonstrated the advancement of deep-learning-based approaches to establish robust variant calling systems. Discrepancies in variant representation between true variants and sequencing alignments can lead to incorrect training labels, impacting the efficacy of learning-based approaches in managing complex variants during real calling scenarios. Calling variants in functional and low-complexity regions like the major histocompatibility complex, homopolymer, and tandem repeat regions [12] poses challenges that can be mitigated through the unification of complex variant representations. DeepVariant [9] incorporates a haplotype labeler function to verify variant-alignment consistency before model training. However, this method is constrained by the searching window size and does not consider haplotype information. On the other hand, Clair3 [10] utilized unified variants rather than GIAB truth variants for constructing training datasets, excluding variants lacking read support, while this approach is computationally intensive and time-consuming for practical usage.

Given these challenges, this study aims to address the demand for accurate representation unification of small variant calls. In this study, we proposed Repun, the first approach to achieve representation unification without relying on the variant call results. Specifically, Repun takes sequencing alignments and variants as input to maximize equivalent variant representation while minimizing the presence of missing variants. To handle the complex matching cases more efficiently, we proposed a haplotype-aware matching strategy to reduce massive possible variant-candidate haplotype comparisons. We utilized the heterozygous variants to reconstruct phased haplotype information and associate the variants with the phased alignments to enhance match accuracy. The selection of the optimized unified pair was based on criteria such as fewer missing variants and a greater number of read alignments supporting the unified outcomes. We demonstrated that the proposed Repun algorithms worked properly in both long-read and short-read platforms, including Oxford Nanopore Technology (ONT), Pacific Biosciences (PacBio), and Illumina platforms in various scenario settings. Extensive benchmarking across multiple GIAB samples demonstrates that Repun successfully unified >99.5% of variants with high sensitivity nearly without introducing false signals.

## Result

### Overview of Repun

**Figure 1:**
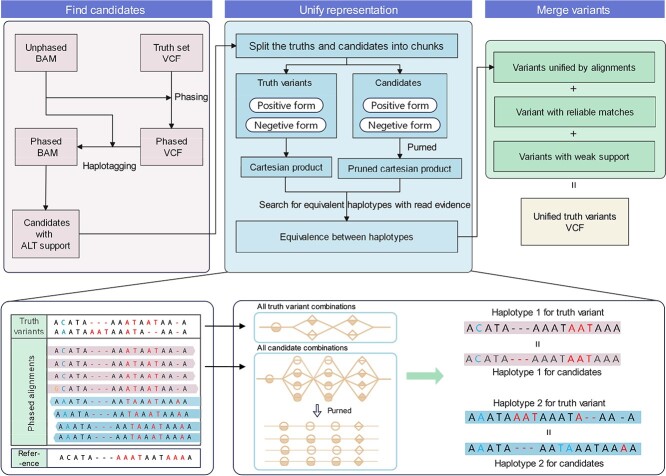
Overview of Repun’s variant representation unification workflow. Repun follows three steps to derive the unified variants in the alignment and outputs them to a VCF file. In Step 1, the heterozygous variants are phased using the variants and sequencing alignment to acquire phased variants and haplotagged BAM. In Step 2, the variants and candidates are split into small chunks to get all possible haplotype representations. A pruned haplotype match strategy was employed to reduce the complexity and find the equivalent haplotype with the maximal read evidence. In Step 3, the variants unified by alignments, variants with reliable matches, and variants with weak support are merged to generate the final output VCF file.

The workflow of Repun is depicted in Fig. 1, Repun is an alignment-based method for small variant representation unification that employs a standardization process for variants in the VCF and candidates in the alignment, to ensure a consistent variant-alignment representation. Repun leverages the heterozygous variants to perform phasing for query alignments, thereby acquiring comprehensive haplotype information for unification. A refined query candidate selection strategy is employed, pairing the query candidates with the corresponding variants (variant-candidate pairs). Repun accelerates the unification process by pruning the combination of variant-candidate pairs based on read evidence and is credited to the optimized pair with the maximum read support. Notably, Repun operates without needing variant calling intervention and can acquire comprehensive variant representation information that is consistent with sequencing alignment.

**Figure 2:**
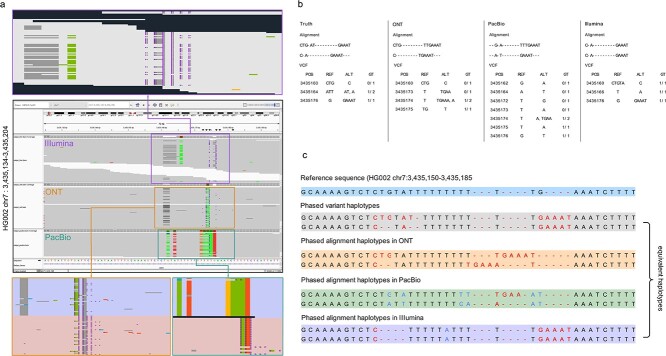
Unified variant representation of three platforms with the equivalent haplotype. (a) the IGV and alignment plots depict a specific region (HG002 chr7:3,435,134-3,435,204), showcasing detailed alignments from different platforms in cropped windows. (b) the variant call files (VCFs) present the variants in VCF and the reported query variants. Each VCF includes the variant position coordinate (POS), reference alleles (REF), alternate alleles (ALT), and genotype (GT). (c) the reference and phased variant haplotypes sequences are displayed for different sequencing platforms. ‘-’ indicates a gap for matching. If we exclude the extending gaps for comparison, all sequences from the three platforms share the equivalent haplotypes.

Figure 2 illustrates an example showcasing the unified variant representation across three platforms, demonstrating equivalent haplotypes. Specifically, the IGV and alignment plots focus on a specific region (HG002 chr7:3,435,134-3,435,204) in GRCh38. In this region, a total of three variants cluster within a 20 bp window, as indicated by the GIAB truths. However, when examining the alignments from the three platforms, it becomes evident that each platform presents a distinct variant representation. Notably, the ONT platform identifies four variants, PacBio identifies seven variants, while Illumina detects three variants. These variant calls are supported by sufficient read evidence specific to each platform. Despite the different variant representations initially observed, all the listed variants ultimately converge to the same representation after resolving the gaps, insertions, and deletions. This indicates that the variants in the alignment accurately represent the variants in VCF while displaying multiple representations across different platforms. The distinct representation requires precise variant representation unification before downstream analysis.

We conducted performance evaluations of Repun on two long-read platforms: PacBio and ONT and one short-read platform: Illumina. Our primary objective was to assess the unification capabilities and robustness of Repun across various platform and sequencing condition settings. Additionally, we aimed to evaluate the influence of enhanced technologies and sequencing chemistries, focusing on the impact of phased haplotype results and error profiles on variant unification performance. A summary of the dataset used for benchmarking is shown in Table 1 and Supplementary Notes.

**Table 1:** A summary of the datasets used for unification and evaluation.

Platform	Sample	Reference	Aligner	Coverage	Source	Basecaller/Chemistry/Instruments
ONT	HG002	GRCh38	minimap2	64.0	-	Guppy v2.3.8
	HG002	GRCh38	minimap2	55.0	GIAB	Guppy v3.2.4
	HG002	GRCh38	minimap2	49.0	precisionFDA	Guppy v3.6.0
	HG002	GRCh38	minimap2	120.0	HPRC	Guppy v4.2.2
	HG002	GRCh38	minimap2	119.0	HPRC	Guppy v5.0.16
	HG002	GRCh38	minimap2	79.0	EPI2ME labs	Dorado v0.3.0 4 kHz
	HG002	GRCh38	minimap2	91.2	EPI2ME labs	Dorado v0.3.0 5 kHz
	HG003	GRCh38	minimap2	84.8	precisionFDA	Guppy v3.6.0
	HG004	GRCh38	minimap2	87.4	precisionFDA	Guppy v3.6.0
PacBio	HG002	GRCh38	pbmm2	32.0	GIAB	CCS-11 kb, chemistry 2.0
	HG002	GRCh38	pbmm2	31.0	GIAB	CCS-15 kb, chemistry 2.0
	HG002	GRCh38	pbmm2	53.3	GIAB	CCS-20 kb, chemistry 2.0
	HG002	GRCh38	pbmm2	34.9	precisionFDA	CCS-15 kb, chemistry 2.0
	HG003	GRCh38	pbmm2	34.3	precisionFDA	CCS-15 kb, chemistry 2.0
	HG004	GRCh38	pbmm2	34.3	precisionFDA	CCS-15 kb, chemistry 2.0
Illumina	HG002	GRCh38	BWA-MEM	38.8	precisionFDA	NovaSeq 6000
	HG002	GRCh38	BWA-MEM	50.0	Google HealthGenomics	HiSeqX
	HG003	GRCh38	BWA-MEM	39.1	precisionFDA	NovaSeq 6000
	HG003	GRCh38	BWA-MEM	50.0	Google HealthGenomics	HiSeqX
	HG004	GRCh38	BWA-MEM	39.3	precisionFDA	NovaSeq 6000
	HG004	GRCh38	BWA-MEM	50.0	Google HealthGenomics	HiSeqX

### Performance on the ONT platform

For the ONT platform, we compared the ONT datasets with two ONT-provided basecallers, Guppy [13] and Dorado [14], with three sequencing chemistries R9.4.1, R10.4, and R10.4.1 [15] for evaluation. We selected three GIAB samples from HG002-HG004 trio to evaluate the performance of Repun. For the Guppy basecaller, we selected four different official releases by ONT, which are the dataset base-called with Guppy 2.2.3 using the Flip-Flop model, Guppy 3.2.4 HAC(High accuracy) model, Guppy 3.6.0 HAC model, and Guppy 5.0.14 SUP(Super accurate) model. For the Dorado basecaller, we selected two official releases, Dorado v0.3.0 4 kHz and Dorado v0.3.0 5 kHz basecaller models for evaluation.

Figure 4a and Table 3a illustrate the performance achieved by Repun, particularly since Guppy 3.2.4, with precision performance in the GIAB HG002 sample [16] exceeding 99.9%. The recall of variants has also seen improvements with advancements in basecalling and sequencing chemistry. Starting from Guppy v5.0.16 basecaller version with R10.4 chemistry, the recall has reached 99.90% for SNP variants and 99.59% for Indel variants. Notably, the performance for Indel variants exhibited a more significant improvement from R9.4.1 chemistry to R10.4 chemistry, with Repun achieving 99.99% precision, 99.59% recall, and 99.79% F1-score in R10.4 chemistry. This reflects a 0.59% recall improvement compared to R9.4.1 chemistry while using the same basecaller version.

Furthermore, in more recent developments, Dorado has replaced Guppy as the default basecaller since proposing R10.4.1 chemistry, resulting in improved read accuracy. The recall for both SNP and Indel variants continues to improve, reaching 99.98% for SNPs and 99.64% for Indels. Additionally, in the fourth quarter of 2023, ONT released a flowcell with a higher sampling frequency and improved throughput, increasing the sampling frequency from 4 kHz to 5 kHz. It is noteworthy that while the performance for SNPs remained consistent, the Indel recall improved from 99.64% to 99.80%. The results underscore the ability of Repun to accurately capture variants with high specificity, as demonstrated by the precision achieved across different basecalling versions and chemistries.

### Performance on the PacBio platform

**Figure 3:**
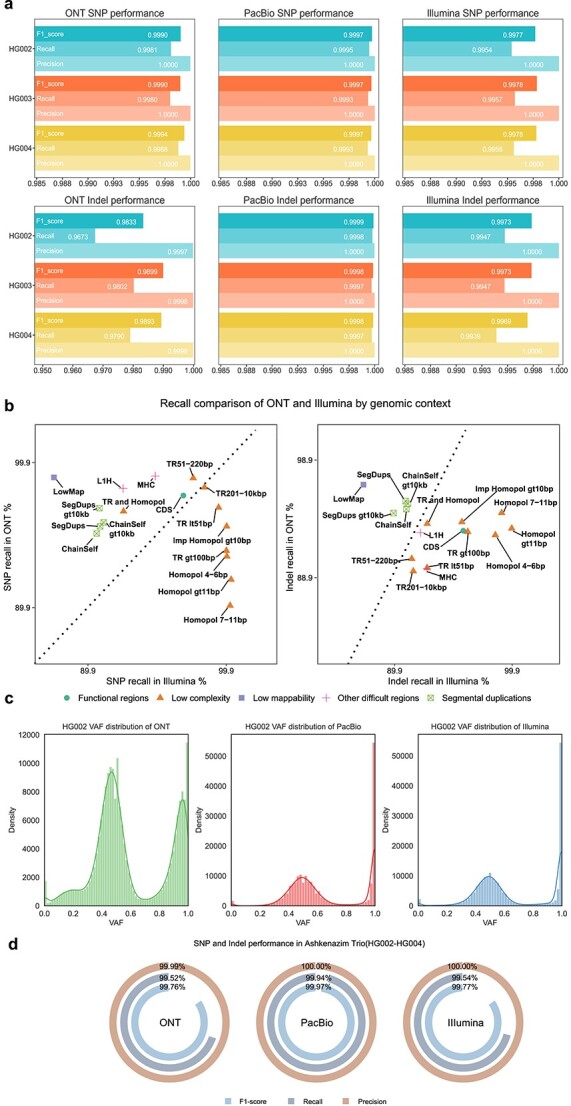
Benchmarking results of Repun in different sequencing platforms. (a) the SNP and Indel performance of the Repun in three different platforms for the HG002, HG003, and HG004 samples. (b) a performance comparison between the ONT and Illumina platforms based on genomic context using the 30x HG002 dataset. The stratification category result was generated using GIAB stratification v2 and hap.py. Both the x-axis and y-axis are presented in a logarithmic scale. Dots above the diagonal line indicate better performance of the ONT platform compared to the Illumina platform. (c) the variant allele frequency (VAF) distribution of the GIAB HG002 sample in three platforms. The variants were collected from the GIAB v4.2.1 truth set VCF, and the VAF values were calculated from the BAM files obtained from the PrecisionFDA truth challenge V2. The PacBio and Illumina datasets exhibit a sharp peak at 0.5 and 1.0, respectively, while the ONT dataset shows a smooth VAF distribution ranging from 0.8 to 1.0. (d) the overall SNP and Indel performance of the Ashkenazim trio across three sequencing platforms. PacBio demonstrates the best F1-score performance, while ONT and Illumina exhibit comparable F1-score performance.

For the PacBio platform, we benchmarked PacBio single-molecule CCS reads [17] with high accuracy averaging up to 20 kb with a read accuracy of over 99.9%. For our analysis, we utilized PacBio CCS datasets with three average read lengths: CCS 11 kb, CCS 15 kb, and CCS 20 kb. The dataset was generated by different sources, including PrecisionFDA Truth Challenge V2 [18] and Google Health Genomics [19], with more details of datasets described in Table 1. As shown in Table 3b, we observed that the PacBio CCS dataset achieved >99.96% SNP F1-score and > 99.97% Indel F1-score across all three chemistries in ~30x coverage settings. Moreover, by leveraging high-quality sequencing data, Repun accurately identifies nearly all (>99.92%) true variants present in the provided datasets. We also benchmark the performance in Ashkenazim Trio (HG002-HG004) provided by the PrecisionFDA Truth Challenge V2, all the performances in the trio have an average 99.7% SNP F1-score and 99.8% Indel F1-score, as shown in Fig. 3d**and**Table 2.

**Table 2:** Benchmark result in Ashkenazim Trio in PrecisionFDA Truth Challenge V2 dataset.

Platform	Sample	Overall			SNP			Indel			Insertion			Deletion		
		Precision	Recall	F1	Precision	Recall	F1	Precision	Recall	F1	Precision	Recall	F1	Precision	Recall	F1
ONT	HG002	99.99%	99.39%	99.69%	100.00%	99.81%	99.90%	99.97%	96.73%	98.33%	100.00%	96.29%	98.11%	99.95%	97.13%	98.52%
	HG003	100.00%	99.56%	99.78%	100.00%	99.80%	99.90%	99.98%	98.02%	98.99%	100.00%	97.95%	98.96%	99.96%	98.07%	99.01%
	HG004	99.99%	99.62%	99.81%	100.00%	99.88%	99.94%	99.98%	97.90%	98.93%	100.00%	97.85%	98.91%	99.97%	97.95%	98.95%
PacBio	HG002	100.00%	99.94%	99.97%	100.00%	99.93%	99.97%	100.00%	99.97%	99.98%	100.00%	99.97%	99.99%	100.00%	99.97%	99.98%
	HG003	100.00%	99.94%	99.97%	100.00%	99.93%	99.97%	100.00%	99.97%	99.98%	100.00%	99.97%	99.99%	100.00%	99.96%	99.98%
	HG004	100.00%	99.95%	99.97%	100.00%	99.95%	99.97%	100.00%	99.98%	99.99%	100.00%	99.98%	99.99%	100.00%	99.98%	99.99%
Illumina	HG002	100.00%	99.54%	99.77%	100.00%	99.56%	99.78%	100.00%	99.39%	99.69%	100.00%	99.15%	99.57%	100.00%	99.61%	99.80%
	HG003	100.00%	99.56%	99.78%	100.00%	99.57%	99.78%	100.00%	99.47%	99.73%	100.00%	99.23%	99.61%	100.00%	99.68%	99.84%
	HG004	100.00%	99.53%	99.76%	100.00%	99.54%	99.77%	100.00%	99.47%	99.73%	100.00%	99.25%	99.62%	100.00%	99.67%	99.83%

### Performance on the Illumina platform

We benchmark Repun performance in the short-read dataset using the HG002-HG004 Ashkenazim Trio dataset, which is acquired from the PrecisionFDA Truth Challenge V2. The Illumina dataset was generated using the Illumina NovaSeq 6000 system, employing a 2x151 bp high PCR-free library. The results in Fig. 3a and Fig. 3d show that the Illumina dataset achieved >99.78% SNP F1-score and > 99.69% Indel F1-score in the Ashkenazim Trio dataset with ~30x coverage settings.

We also compared Repun’s performance on different sequencing instruments, the NovaSeq 6000 system and the HiSeqX systems, with the dataset generated by Google Health Genomics [19]. As depicted in Table 3c, the NovaSeq 6000 system outperformed the HiSeqX systems in consolidating the variants, exhibiting an average SNP F1-score of 99.79% compared to 99.72%, and an Indel F1-score of 99.75% versus 99.71% in GIAB Ashkenazim Trio. Due to the limitations imposed by the read length, the ability to reliably match variants in short-read datasets is hindered, making phasing less feasible. Compared to the results obtained from long-read datasets, Illumina exhibits a lower recall in capturing true variants, primarily attributed to its inability to accurately map and identify variants in challenging low-mappability and low-complexity regions.

**Table 3:** Benchmark results in different sequencing platforms. (a) Performance on the ONT platform across various chemistries and basecaller versions. Illustration IGV of variant in VCF and query candidates. (b) Performance on the PacBio platform across different sequencing templates. (c) Performance on the Illumina platform across different sequencing instruments.

Sample	Chemistry	Basecaller	Overall			SNP			Indel		
			Precision	Recall	F1	Precision	Recall	F1	Precision	Recall	F1
ONT HG002 dataset	R9.4.1	Guppy v2.3.8	99.99%	95.03%	97.45%	100.00%	99.13%	99.56%	99.92%	68.80%	81.49%
	R9.4.1	Guppy v3.2.4	99.99%	99.02%	99.50%	100.00%	99.70%	99.85%	99.98%	94.66%	97.25%
	R9.4.1	Guppy v3.6.0	99.99%	99.39%	99.69%	100.00%	99.81%	99.90%	99.97%	96.73%	98.33%
	R9.4.1	Guppy v5.0.16	99.99%	99.79%	99.89%	99.99%	99.91%	99.95%	99.95%	99.00%	99.47%
	R10.4	Guppy v5.0.16	99.99%	99.90%	99.94%	99.99%	99.94%	99.97%	99.99%	99.59%	99.79%
	R10.4.1	Dorado v0.3.0 4 kHz	100.00%	99.93%	99.96%	100.00%	99.98%	99.98%	99.99%	99.64%	99.81%
	R10.4.1	Dorado v0.3.0 5 kHz	100.00%	99.95%	99.97%	100.00%	99.97%	99.98%	99.99%	99.80%	99.90%

### Repun result reveals the findings across platforms

We compare the Repun’s result across three sequencing platforms in the datasets used in PrecisionFDA Truth Challenge V2 [18]. As shown in Fig. 3a, Fig. 3d and Table 3, we noticed that PacBio achieved the best recall and precision among the three platforms, with an average 99.97% SNP F1 score and 99.98% F1 score in the Ashkenazim trio. ONT has a better SNP performance than Illumina, with a 99.92% F1 score versus 99.78%, and inferior Indel performance than Illumina, with a 98.96% F1 score versus 99.73% F1 score. We tried to split the variants into various genome stratifications to further compare the difference between the ONT and Illumina datasets. As shown in Fig. 3b, the SNP performance indicates ONT can detect more variants in complex regions, such as segmental duplication regions and functional regions. The low complexity regions such as homopolymer and tandem repeat are still the main challenges for ONT to detect the variant correctly in those regions. However, with the advancements in ONT sequencing technologies, the read accuracy of ONT has undergone significant improvements. The performance of Repun on the latest ONT data utilizing R10.4.1 chemistry and Dorado 5 kHz sequencing has achieved a SNP F1-score of 99.98% and an Indel F1-score of 99.90%. These results surpass the datasets utilized in the PrecisionFDA Truth Challenge V2, where the basecaller version was Guppy v3.6.0 and the sequencing chemistry was R9.4.1.

### Computation time analysis w/o haplotype pruning

We conducted a comparative analysis of the Repun results with and without haplotype pruning. The introduction of haplotype pruning, along with read evidence, significantly reduced the overall matching complexity from a naive Cartesian product of all variants and query candidates to a linear relationship with the coverage of read evidence. Specifically, using four 12-core Intel Xeon Silver 4116 processors, the computational time exhibited improvements for different sequencing technologies. The computational time for the unification was reduced from ~150 minutes to ~5 minutes for the ONT dataset, it decreased from ~60 minutes to ~2 minutes for the PacBio dataset. Furthermore, in the case of Illumina, the computational time was reduced from ~110 minutes to ~30 minutes. Notably, the overall computation time of long-read unification time is lower than the short-read dataset, mainly because more alignment could be haplotagged in long-read to reduce the combination searching in unification. As depicted in Fig. 3b, the alignment rate of haplotagged reads compared to the total alignment rate is 44.9% and 73.2% for the ONT and PacBio platforms. This percentage is significantly higher compared to the 7.7% alignment rate on the Illumina platform, underscoring the need for phased alignment to expedite the unification process.

### Unified result analysis in various platforms

We compared the Repun unified result in three platforms using the ~30x HG002 dataset. As shown in Fig. 3c and Table 4, we used the GIAB HG002 truth variants and selected the variants in GIAB high-confident BED for evaluation. The total number of truth variants is 3 890 596, where there are 3 365 127 SNPs and 525 469 Indels. The candidates exceeding the minimal allele frequency range from 4 439 684 to 6 719 985. By site-to-site comparison with truth variants, we identified ~2.5 million that are reliably matched and thus were masked from unification. The remaining candidates were fed into the unification workflow. After unification, there are 50 053, 7299, and 38,533 variants that were considered with low read evidence support and were rescued for position alternate allele match. After the whole unification process, there are still ~2 k truths with insufficient read evidence for long-read platforms and with ~17 k variants in the Illumina platforms. More missing truths indicate that the variants missed in short-read platforms might be the variants in low-complexity regions that Repun cannot examine due to lacking coverage support. Moreover, the different representations between alignments and truth variants could confuse the model training for deep learning-based variant caller due to incorrect training label without representation unification. We employed Repun to generate unified result to train Clair3 models. In our benchmarks, the representation unification alone generally increased SNP recall by ~0.2% and Indel recall by ~2% using the model trained on ONT Guppy4 dataset.

**Table 4:** Unified result analysis in different platforms.

Sample	Platform	Total candidates	Total truths	Reliable match (truths)	Truths to be unified	Truths rescued by RU	Truths with insufficient evidence
HG002 ~ 30x	ONT Dorado v0.3.0 5 kHz	44,39,684	38,90,596	27,92,728	10,45,533	50,053	2282
	PacBio CCS-20 kb, chemistry 2.0	74,15,236	38,90,596	25,14,339	13,66,499	7299	2459
	Illumina NovaSeq 6000	67,19,985	38,90,596	28,35,877	9,98,194	38,533	17,992

## Methods

### Overview of Repun

Figure 1 shows an overview of Repun’s representation unification workflow. Starting from the alignments in the BAM/CRAM format of an input sample, Repun follows three steps to derive the unified variants in the alignment and output them to a VCF file. In Step 1, the heterozygous variants are phased using the query BAM to acquire a phased zygosity. We then scan the alignment to identify all potential candidates that are eligible for unification. Moving on to Step 2, we focus on identifying reliable variants that exhibit consistency between haplotypes and the alignment. These candidates are unified with the variants, resulting in the acquisition of the desired haplotype representation. To optimize this process, we introduce a pruned haplotype match strategy that leverages read evidence to reduce the possible variant-candidate pairs, thereby achieving an optimized unification. Additionally, we incorporate a rescue mechanism for low-allele frequency variants with weak support that were initially overlooked during unification. Finally, in Step 3, the variants unified by alignment, variants with reliable matches, and variants with weak support are concatenated to generate the final output VCF file.

### Repun’s input and output

Input. Repun takes the input mainly includes two components: 1) a variant to be unified, prepared by authorized sources or other sequencing platforms, and 2) a sequencing alignment with the same reference material with 1). Repun will automate the unification pipeline and get the Output, as illustrated below.

Output. Repun output VCF format output with three category variants: variants with reliable match, unified variants, and variants with weak support. Variants are marked ‘PASS’ or ‘LowQual’ if the allele frequency of the given variant is low (i.e., AF < 0.08, configurable by option). For each variant, the unified reference and alternate alleles, and the unified genotype that are consistent with the alignment are shown.

### Representation unification workflow

#### Variant phasing

Accurate phasing of variants is crucial for obtaining comprehensive haplotype information. A heterozygous variant recorded in a single haplotype is deemed more reliable than a sequencing error affecting both haplotypes. We adopt WhatsHap [20] for variant phasing and disable the ‘—distrust-genotypes’ option. This enables the switching of variant genotypes in the event of missing variants, minimizing the minimum error correction (MEC) cost [21] between the variant and the sequencing alignment. Enabling this option led to a reduction of ~10% MEC during the variant phasing step.

#### Alignment haplotagging

The alignments are then haplotagged by leveraging the phased variants to identify reads corresponding to the sample haplotype. The phasable read is designated with the ‘HP:x’ tag, where ‘x’ indicates the specific haplotype assigned in the haplotagging process. Phased alignment is crucial for variant unification, simplifying the matching process efficiently. We also introduced an option ‘—disable_phasing’ to disable phasing to facilitate unification in amplicon and target sequencing data or non-human sequencing samples.

#### Identify candidates for unification

The initial step involves identifying candidates within the sequencing alignment. For SNP variants, a minimum alternate allele frequency of 0.08 is enforced for the PacBio and Illumina platforms, and 0.15 for the ONT platform. For Indel variants, a minimum alternate allele frequency of 0.15 is permitted. Furthermore, a minimum coverage threshold of 4, determined through manual selection, is employed as an additional criterion.

#### Mask reliable matches

**Figure 4:**
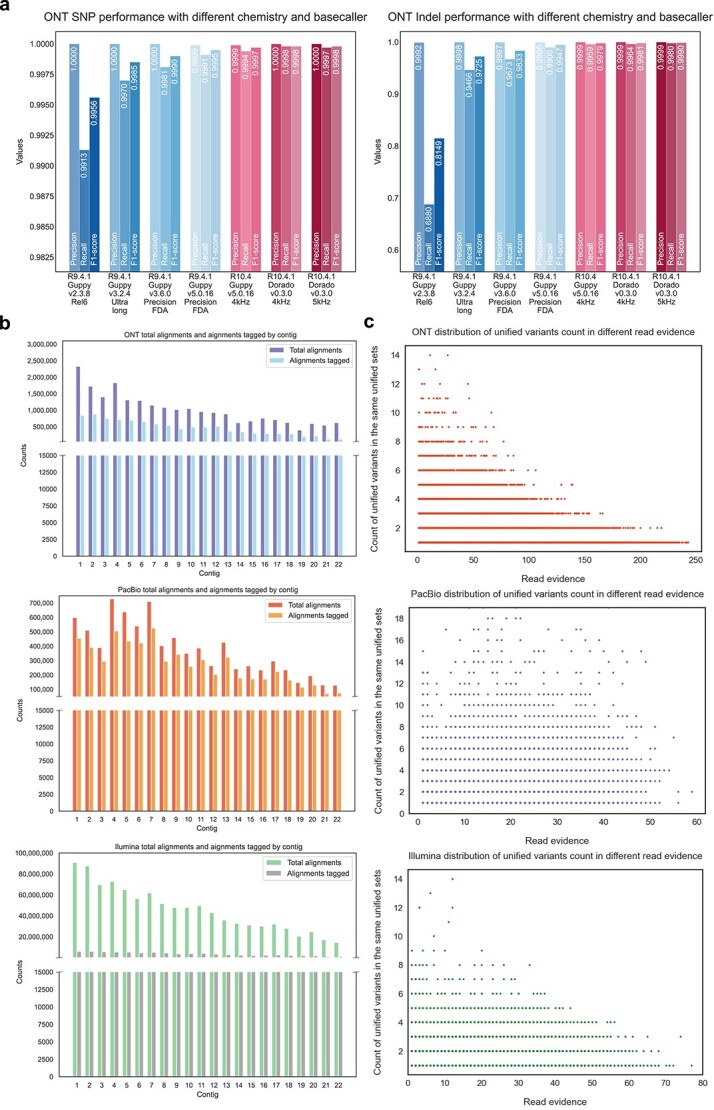
Performance analysis of Repun under different sequencing settings. (a) the performance of Repun on the ONT platform across various chemistries and basecaller versions. (b) Distribution of the total alignment and haplotagged alignments from the HG002 dataset. (c) Scatter plots illustrate the relationship between read evidence of unified variants and missing variants in the different datasets.

Before consolidating a great number of candidates with the true variants, a site-to-site verification is applied for each variant and its corresponding candidate in the alignment. A variant-candidate match is deemed reliable when the alternate allele frequency is between 0.5-ε and 0.5 + ε for a heterozygous variant or greater than 1-ε for a homozygous variant, where ε is a tolerance parameter and set as 0.1 by default. As illustrated in Fig. 4b, the reliable matches result in the exclusion of 71.8%, 64.6%, and 72.9% of variants in ONT, PacBio, and Illumina platforms, respectively.

#### Haplotype generation for variants and candidates

The objective of representation unification is to achieve a haplotype match between candidates and variants with minimal missing variants and maximal read support for the haplotype match. For each variant, two forms can be represented: positive form and negative form. Positive form means the given variant matches with alignment with the expressed reference base, alternative base, and genotype representation. A negative form allows the variant to be missing. Suppose there are *n* variants for unification, and *i* represents the *i*-th variant, where $\in \left[0,n\right)$. ${V}_i^T$ and ${R}_i^T$ represents the positive and negative form of the *i*-th variant, respectively. All possible variant haplotypes can be derived by taking the Cartesian product of the two forms of each variant, denoted as *S_Truth_*, whereas


(1) \begin{equation*}\begin{array}{l} {S}_{Truth}=\left\{\left({S}_0,{S}_1,\dots, {S}_{n-1}\right)\left|{S}_0\in \left\{{R}_0^T,{V}_0^T\right\},\right.\right.\\ \left.{S}_1\in \left\{{R}_1^T,{V}_1^T\right\},\dots, {S}_{n-1}\in \left\{{R}_{n-1}^T,{V}_{n-1}^T\right\}\right\} \end{array}\end{equation*}

*s_i_* indicates that the status of the *i*-th variant. For simplicity, we denote *S_Truth_* as


(2) \begin{equation*} {S}_{Truth}=\prod \limits_{i=0}^{n-1}\left\{\left\{{R}_i^T,{V}_i^T\right\}\right\} \end{equation*}

Assume that there are *m* candidates for unification and *j* represents the *j*-th candidate, where $j\in \left[0,m\right)$. The possible candidate status falls into three categories: homozygous reference, heterozygous variant, and homozygous variant. ${R}_j^C$ and ${V}_j^{homo}$ represent homozygous reference and homozygous variant forms of the *j*-th candidate, respectively. ${V}_j^{het01}$ and ${V}_j^{het10}$ signify two phased heterozygous variant forms. All possible candidate haplotypes *S_candidates_* is determined by considering the Cartesian product of the candidates, as defined below:


(3) \begin{equation*} {S}_{candidates}=\left\{\left({S}_0,{S}_{1,\cdots, }{S}_{m-1}\right)\left|\begin{array}{c}{S}_0\in \left({R}_0^C,{V}_0^{het01},{V}_0^{het10},{V}_0^{homo}\right)\\{}{S}_{m-1}\in \left({R}_{m-1}^C,{V}_{m-1}^{het01},{V}_{m-1}^{het10},{V}_{m-1}^{homo}\right)\end{array}\right.\!\!\!\!\!\right\} \end{equation*}

For simplicity, we denote defined *S_candidates_* as:


(4) \begin{equation*} {S}_{candidates}=\prod \limits_{j=0}^{m-1}\left\{\left\{{R}_j^C,{V}_j^{het01},{V}_j^{het10},{V}_j^{homo}\right\}\right\} \end{equation*}

Then we rely on *S_Truth_* and *S_candidates_* to acquire an optimized haplotype match.

#### Variant-candidate combination pruning with read evidence

Finding the best match from all possible variant-candidate combinations is often computationally intensive, particularly with large candidate sets. The computation complexity of matching grows rapidly as the number of candidates increases. To address this, we propose a strategy that focuses on combinations supported by sequencing reads. Figure 4c demonstrates that variants in three sequencing platforms can be clustered based on read evidence to reduce computational complexity and achieve unification. We observed that as the number of variants within a specific flanking window increased, the read evidence decreased while maintaining the accuracy of haplotypes with the variants across all three platforms.

**Figure 5:**
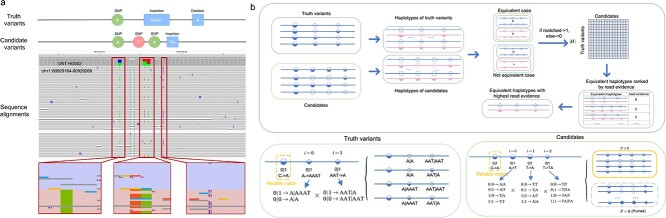
Illustration of the haplotype generation and pruning mechanism. (a) the illustration IGV of variants in VCF and query candidates. (b) Illustrating the process of variant haplotype and candidate haplotype generation, followed by variant-candidate haplotype matching to obtain equivalent haplotype representation. Haplotypes lacking read evidence were pruned from the comparison.

The combination pruning workflow is shown in Fig. 5. Assuming that the total candidate Cartesian haplotype size is x, and the total alignment overlapping the candidates is *y*. Then the likelihood of each haplotype scoring is denoted as ${P}_a=x/y$. We removed haplotypes with *P_a_* equal to 0 to exclude combinations missing in the alignments, resulting in a refined set of pruned candidates’ haplotypes as follows:


(5) \begin{equation*} {S}_{candidates}=\left\{\left({S}_0,{S}_{1,\cdots, }{S}_{m-1}\right)\left|\begin{array}{c}{S}_0\in \left({R}_0^C,{V}_0^{het01},{V}_0^{het10},{V}_0^{homo}\right)\\{}\begin{array}{l}{S}_{m-1}\in \left({R}_{m-1}^C,{V}_{m-1}^{het01},{V}_{m-1}^{het10},{V}_{m-1}^{homo}\right)\wedge \\{}\kern3em P\left({S}_0,{S}_{1,\cdots, }{S}_{m-1}\right)>0\end{array}\end{array}\right.\!\!\!\!\!\!\!\!\!\right\} \end{equation*}

#### Maximizing the read evidence matrix for matching

Upon obtaining all possible haplotypes with read evidence, a pairwise comparison is performed. The core concept is the equivalence of variant haplotype representation with candidate haplotype, which might even differ at individual candidate sites. We set up an equivalent matrix *K*, where the element *K_ij_* of the matrix represents the comparison result of the *i*-th *variant* haplotype *S_Ti_* and the *j*-th haplotype *S_Cj_* in the candidate haplotype. Then the element is labeled as below:


(6) \begin{equation*} {K}_{ij}=\left\{\begin{array}{c} \!\!\!\!\!\! True\kern1em {S}_{Ti}={S}_{Cj}\\{}\!\!\!\! False\kern1em {S}_{Ti}={S}_{Cj}\end{array}\!\!\!\!\right\} \end{equation*}

An equivalent haplotype set *E* were then calculated from all matching haplotypes, represented as:


(7) \begin{equation*} E=\left\{\left({e}_0,{e}_{1,\dots, }{e}_{\phi}\right)\left|\left({e}_0,{e}_{1,\dots, }{e}_{\phi}\right)\right.\in{S}_{Truth}\wedge \left({e}_0,{e}_{1,\dots, }{e}_{\phi}\right)\in{S}_{Candidates}\right\} \end{equation*}

After acquiring the set of equivalent haplotypes, the corresponding haplotypes with the highest read support are identified and those candidates are included in the unified output.

#### Computation complexity analysis for variant-candidate matching

The maximum time complexity of this step is *O(2^n^)* for the variant haplotype generation, and *O(4^m^)* for the candidate haplotype generation. Assuming that the average comparison time between each variant and alignment in the set is *a*, the time complexity before pruning is$O\left({2}^n\times{4}^m\times a\right)$. After applying pruning, the time is reduced to $O\left({2}^n\times w\times a\right),$ where *w* stands for the size of candidate haplotype size after operation, and *w* correlates directly with the read coverage of the benchmarking regions. Notably, the implementation of combination pruning demonstrates a significant computational reduction.

### Benchmarking metrics

Though Repun can produce unified results directly for variant evaluation, we used hap.py as a fair metrics benchmarking tool, as it provides a complete metrics definition. We used hap.py to compare the unified variants against the truth variants and used Repun’s ‘GetOverallMetrics’ module to generate three metrics, ‘Precision’, ‘Recall’, and ‘F1-score’, for five categories: ‘Overall’, ‘SNP’, ‘Indel’, ‘Insertion’, and ‘Deletion’. For the true positives (TP), we considered the variants and genotypes that matched the truth and query (*QUERY.TP* and *TRUTH.TP*). False positives (FP) were defined as variants that had mismatching genotypes or alternate alleles in regions of a truth set (*QUERY.FP*). False negatives (FN) were defined as variants present in the truth set but missed in the query (*TRUTH.FN*). From the number of *QUERY.TP*, *QUERY.FP*, *TRUTH.TP*, and *TRUTH.FN* and hap.py, we compute the three metrics ‘Precision’, ‘Recall’, and ‘F1-score’ as:


(8) \begin{equation*} Precision=\frac{QUERY. TP}{QUERY. TP+ QUERY. FP} \end{equation*}

(9) \begin{equation*} Recall=\frac{TRUTH. TP}{TRUTH. TP+ TRUTH. FN} \end{equation*}

(10) \begin{equation*} F1- score=\frac{2^{\ast }{Precision}^{\ast } Recall}{Precision+ Recall} \end{equation*}

We used qfy.py with GIAB genome stratifications V2.0 to evaluate Repun’s performance in challenging genome regions.

### Computational performance

Repun was implemented with Python and leveraged PyPy for speedup. For a typical human whole-genome-sequencing sample with 30-fold coverage, Repun takes ~90 minutes for ONT, ~40 minutes for PacBio, and ~ 100 minutes for Illumina data, using four 12-core Intel Xeon Silver 4116 processors. The unification process was completed in ~5 minutes for the long-read dataset and ~ 30 minutes for the short-read dataset, with major computational time allocated to massive candidate discovery, where the duration varied based on the error rates of the sequencing alignment. The memory consumption is capped at 500 MB per process. Repun supports automatically splitting the genome into small user-customized chunks, performing candidate discovery and variant unification in massive parallelization for individual chunks, and merging the results into the final VCF output.

## Discussion

Diverse sequencing technologies, chemistries, and upstream sequencing settings contribute to variations in variant representation. Research institutions such as Real Time Genomics and the GA4GH Benchmarking Team have made significant advancements in developing variant unification methods and standards for comprehensive small germline variant evaluation. However, the evaluation approaches such as RTG tools and hap.py are designed for evaluating VCF results and cannot acquire the complete variant status before variant calling.

Variant representation unification is crucial for generating accurate ‘sequence-aligned’ variants for model training and capturing haplotypes within complex genomic regions. In this study, we proposed Repun, an approach aimed at unifying variant to be consistent with the alignment. We employed various techniques such as haplotype-aware candidate discovery, reliable variant masking, and candidate haplotype pruning to get comprehensive unified outcomes. Our benchmarking results demonstrate that our approach can effectively unify variants with high sensitivity across multiple long-read and short-read datasets.

Moreover, Repun presents a significant capacity to access variant accuracy in emerging technologies without explicit variant calling. This capability opens new avenues for leveraging Repun’s strengths in accurately identifying variants, thereby providing valuable insights into the sequencing conditions and providing confident accordance for downstream pipelines, such as haplotype generation, sequencing quality assessment, etc.

One limitation of this work is that although Repun demonstrates the capability to unify variants with allele frequency as low as 0.08, it may overlook some extremely low-frequency variants with complex representations during unification, limiting its applicability in phasing and unification for these variants. To overcome this challenge, a local-assembly approach becomes imperative in our ongoing work. Local-assembly aims to modify and rectify the aligned variants, particularly benefit Indel variants in low-complexity and difficult-to-map regions. By doing so, we aim to ensure a more comprehensive variant unification.

**Key Points**

- The tool is designed for accurate variant representation unification of small variants without a variant calling process.
- The tool supports efficient variant-alignment unification in short-read and long-read platforms with high sensitivity under various sequencing scenarios.
- We introduce a haplotype-aware variant-alignment matching strategy to obtain comprehensive variant haplotype representation.
- The tool is a crucial solution for deep-learning-based variant calling truth generation and sequencing quality assessment.

## Supplementary Material

Repun_supplementary_bbae613

## Data Availability

The (i) links to the reference genomes, benchmarking materials, and ONT, PacBio, and Illumina data, and (ii) the benchmarked commands and parameters used in this study are available in the Supplementary Notes. All analysis results, including the VCFs and other analysis output, are available at http://www.bio8.cs.hku.hk/repun/analysis_result.

## Code availability

Repun is open source and available at https://github.com/zhengzhenxian/repun under the BSD 3-Clause license. A public Docker image hkubal/repun:latest that can evaluate Repun’s evaluation pipeline is also available for simplicity. Users can run the workflow in one command with the input BAM, VCF, and reference file available. Repun provides user interface flexibility that supports parallel computing, user-customed parameter settings, and workflow refinement.
