# Supplementary material for: Repun: an accurate small variant representation unification method for multiple sequencing platforms
Source: Brief Bioinform. 2024 Nov 25;26(1):bbae613. doi: 10.1093/bib/bbae613 (PMC11586763; doi:10.1093/bib/bbae613)
Supplement: Repun_supplementary_bbae613 [file repun_supplementary_bbae613.docx]

**Repun: An accurate small variant representation unification method for multiple sequencing platforms**

**Supplementary Notes**

# Command

### Read alignment

#### **Minimap2 (v2.17-r941)**

# Align ONT reads using minimap2 to GRCh38 by default

minimap2 -t ${THREADS} -aL -z 600,200 -x map-ont ref.fa input.fastq.gz | samtools view -bh -o output.unsorted.bam -

samtools sort -@${THREADS} -o output.sorted.bam output.unsorted.bam && samtools index -@ ${THREADS} output.sorted.bam

#### **BWA-MEM(v0.7.17-r1188)**

# Align Illumina reads using BWA-MEM with ALT-aware manner by default

bwa mem -t ${THREADS} ref.fa input.R1.fastq.gz input.R2.fastq.gz

### BAM subsampling

#### **Samtools(v1.10)**

samtools view -@ ${THREADS} -s ${RATIO}.${RATIO} -b -o subsampled.bam ${BAM}

samtools index -@ ${THREADS} subsampled.bam

### Coverage calculation

#### **Mosdepth(v0.2.9)**

mosdepth -t ${THREADS} -n -x --quantize 0:15:150: output ${BAM}

### Running Repun variant unification

#### **Repun (v0.1.0)**

docker run -it \

-v ${INPUT_DIR}:${INPUT_DIR} \

-v ${OUTPUT_DIR}:${OUTPUT_DIR} \

zhengzhenxian/repun:latest \

/opt/bin/repun \

--bam_fn ${INPUT_DIR}/sample.bam \

--ref_fn ${INPUT_DIR}/ref.fa \

--truth_vcf_fn ${INPUT_DIR}/truth.vcf \

--threads ${THREADS} \

--platform ${PLATFORM} \ ## options: {ont, hifi, ilmn}

--output_dir ${OUTPUT_DIR}

# Benchmarking

#### **hap.py (v0.3.12)**

hap.py ${GIAB_BASELINE_VCF} output.vcf.gz \

-o ${OUTPUT_DIR}/happy \

-r ${REF} \

-f ${GIAB_CONFIDENT_BED} \

--threads ${THREADS} \

--pass-only \

--engine=vcfeval

# Overall insertion and deletion Precision, Recall, F1-Score

pypy3 ${REPUN} GetOverallMetrics \

--happy_vcf_fn ${OUTPUT_DIR}/happy.vcf.gz \

--output_fn happy.log

#### **qfy.py (v0.3.12)**

# Benchmarking all genome stratifications regions

qfy.py ${OUTPUT_DIR}/happy.vcf.gz \

-t ga4gh \

--stratification v2.0-GRCh38-stratifications.tsv \

-o ${OUTPUT_PREFIX} \

-r ${REF} \

--threads ${THREADS}

# Data availability

### Reference genomes

#### **GRCh38**

ftp://[ftp.ncbi.nlm.nih.gov/genomes/all/GCA/000/001/405/GCA_000001405.15_GRCh38/seqs_for_alignment_pipelines.ucsc_ids/GCA_000001405.15_GRCh38_no_alt_analysis_set.fna.gz](http://ftp.ncbi.nlm.nih.gov/genomes/all/GCA/000/001/405/GCA_000001405.15_GRCh38/seqs_for_alignment_pipelines.ucsc_ids/GCA_000001405.15_GRCh38_no_alt_analysis_set.fna.gz)

#### **GRCh38 Stratification regions (v2.0)**

<https://ftp-trace.ncbi.nlm.nih.gov/giab/ftp/release/genome-stratifications/v2.0/GRCh38/>

### GIAB Truth Variants

#### **HG002 (NA24385), GRCh38, v 4.2.1**

<ftp://ftp-trace.ncbi.nlm.nih.gov/giab/ftp/release/AshkenazimTrio/HG002_NA24385_son/NISTv4.2.1/GRCh38/>

#### **HG003 (NA24149), GRCh38, v 4.2.1**

<ftp://ftp-trace.ncbi.nlm.nih.gov/giab/ftp/release/AshkenazimTrio/HG003_NA24149_father/NISTv4.2.1/GRCh38/>

#### **HG004 (NA24143), GRCh38, v 4.2.1**

<ftp://ftp-trace.ncbi.nlm.nih.gov/giab/ftp/release/AshkenazimTrio/HG004_NA24143_mother/NISTv4.2.1/GRCh38/>

### Oxford Nanopore (ONT) Sequencing Data

#### **HG002 Guppy 3.2.4 (NA24385) , GRCh38**

https://github.com/genome-in-a-bottle/giab_data_indexes/blob/master/AshkenazimTrio/alignment.index.AJtrio_UCSC_ONT_UL_guppy-V3.2.4_2020-01-22.HG002

#### **HG002 Guppy 3.6.0 (NA24385) , GRCh38**

https://precision.fda.gov/challenges/10

#### **HG003 Guppy 4.2.2 (NA24149) , GRCh38**

https://s3-us-west-2.amazonaws.com/human-pangenomics/index.html?prefix=NHGRI_UCSC_panel/HG003/nanopore/Guppy_4.2.2/

#### **HG003 PrecisionFDA (NA24149) , GRCh38**

https://precision.fda.gov/challenges/10

#### **HG004 PrecisionFDA (NA24143) , GRCh38**

https://precision.fda.gov/challenges/10

### Pacific Bioscience (PacBio) HiFi Sequencing Data

#### **HG002 11K (NA24385) , GRCh38**

https://ftp-trace.ncbi.nlm.nih.gov/ReferenceSamples/giab/data/AshkenazimTrio/HG002_NA24385_son/PacBio_SequelII_CCS_11kb/HG002_GRCh38/HG002_GRCh38.haplotag.10x.bam

#### **HG002 20K (NA24385) , GRCh38**

<https://ftp-trace.ncbi.nlm.nih.gov/ReferenceSamples/giab/data/AshkenazimTrio/HG002_NA24385_son/PacBio_CCS_15kb_20kb_chemistry2/GRCh38/HG002.SequelII.merged_15kb_20kb.pbmm2.GRCh38.haplotag.10x.bam>

#### **HG002 PrecisionFDA (NA24385) , GRCh38**

https://precision.fda.gov/challenges/10

#### **HG003 PrecisionFDA (NA24149) , GRCh38**

https://precision.fda.gov/challenges/10

#### **HG004 PrecisionFDA (NA24143) , GRCh38**

https://precision.fda.gov/challenges/10

### Illumina Sequencing Data

#### **HG002 HiSeqX (NA24385) , GRCh38**

https://storage.googleapis.com/brain-genomics-public/research/sequencing/grch38/bam/hiseqx/wgs_pcr_free/40x/HG002.hiseqx.pcr-free.40x.dedup.grch38.bam

#### **HG002 PrecisionFDA (NA24385) , GRCh38**

https://precision.fda.gov/challenges/10

#### **HG003 PrecisionFDA (NA24149) , GRCh38**

https://precision.fda.gov/challenges/10

#### **HG004 HiSeqX (NA24385) , GRCh38**

https://storage.googleapis.com/brain-genomics-public/research/sequencing/grch38/bam/hiseqx/wgs_pcr_free/40x/HG004.hiseqx.pcr-free.40x.dedup.grch38.bam

#### **HG004 PrecisionFDA (NA24143) , GRCh38**

https://precision.fda.gov/challenges/10
